# Elements in scenario‐based simulation associated with nursing students' self‐confidence and satisfaction: A cross‐sectional study

**DOI:** 10.1002/nop2.375

**Published:** 2019-09-27

**Authors:** Camilla Olaussen, Kristin Heggdal, Christine Raaen Tvedt

**Affiliations:** ^1^ Lovisenberg Diaconal University College Oslo Norway; ^2^ The University of Stavanger Stavanger Norway

**Keywords:** active learning, nursing education, self‐confidence, simulation training, student satisfaction

## Abstract

**Aim:**

To identify elements in scenario‐based simulation associated with nursing students' satisfaction with the simulation activity and self‐confidence in managing the simulated patient situation. The study will provide insight to improve the use of simulation as a learning strategy.

**Design:**

A cross‐sectional study.

**Method:**

The Student Satisfaction and Self‐Confidence in Learning scale was used as the outcome measure to identify associations with elements of the Simulation Design Scale and the Educational Practices Questionnaire scale after scenario‐based simulation using patient simulators. First‐year nursing students at a university college in Norway (*N* = 202) were invited to participate and (*N* = 187) responded to the questionnaires.

**Results:**

The mean scores for self‐confidence and satisfaction were 4.16 and 4.57, respectively. In the final multiple linear regression analysis, active learning was associated with satisfaction with the simulation activity, while clear objectives and active learning were associated with self‐confidence in managing the simulated patient situation.

## 1 INTRODUCTION

Scenario‐based simulation using a computerized full‐body‐size patient simulator facilitates the mimicry of real‐life situations (Cato, 2012; Hicks, Coke, & Li, 2009; Shin, Jin‐Hva, & Jung‐Hee, 2015). The use of such simulators may enhance fidelity for many simulated scenarios by enabling the simulation of physiological symptoms for various health conditions as well as physiological reactions to student‐provided care. Whether students feel that simulation experiences mimic real clinical practice is not a fixed property of the patient simulator, however, but also depends on effective simulation design and student engagement (Hamstra, Brygdes, Hatala, Zendejas, & Cook, 2014). Students may be engaged and immersed in the simulated patient scenario by working with the ‘patient' as autonomous clinicians making their own decisions. In this way, students can undergo training in clinical decision‐making and interventions, evaluate health interventions and observe and analyse patient problems (Cant & Cooper, 2010; Cato, 2012; Mills et al., 2014) in a learning environment designed to imitate real patient settings (Hamstra et al., 2014). Simulation is an educational approach that may facilitate student engagement and integrate complex practical and theoretical knowledge (Bland, Topping, & Wood, 2011). The evidence base for such simulations as a learning strategy in nursing education has primarily shown positive outcomes, including self‐confidence and satisfaction, improved knowledge, critical thinking, general competency and clinical skills (Cant & Cooper, 2017; Foronda, Liu, & Bauman, 2013; Haddeland, Slettebø, Carstens, & Fossum, 2018; Merriman, Stayt, & Ricketts, 2014; Shin et al., 2015; Skrable & Fitzsimons, 2014).

## 2 BACKGROUND

Elements of simulation‐based education are described in the National League for Nursing (NLN) Jeffries simulation theory (Jeffries, Rodgers, & Adamson, 2015; Jeffries & Rogers, 2012) The theory provides systematic steps for designing and implementing best‐practice simulation‐based education, and it describes the following elements of the educational practices, simulation design and learner outcomes:

- *Educational practices*: feedback, collaboration, high expectations, active learning, time on tasks, student/faculty interaction and diverse learning experiences.
- *Simulation design*: fidelity, problem‐solving, student support/debriefing and objectives of the simulation.
- *Learner outcomes*: learning, skill performance, critical thinking, learner satisfaction and self‐confidence.

According to the NLN Jeffries simulation theory, educators should consider these elements in planning simulation experiences to achieve high‐level outcomes (Jeffries et al., 2015; Jeffries & Rogers, 2012). The ultimate goal for simulation‐based education is to achieve health outcomes for care recipients. However, to evaluate simulation‐based education, a first step is to examine the students' self‐confidence and satisfaction with the experience.

Instruments for measuring students' self‐confidence and satisfaction and for measuring the presence of elements of the simulation experience that reflect the NLN Jeffries simulation theory have been developed (NLN, 2018). It is already an established opinion that students achieve high scores in self‐confidence and satisfaction (Cant & Cooper, 2017; D'Souza, Arjunan, & Venkatesaperumal, 2017; Lapkin, Levett‐Jones, Bellchambers, & Fernandez, 2010). While students report that they are generally satisfied with and achieve self‐confidence from simulation experiences (Foronda et al., 2013; Haddeland et al., 2018; Merriman et al., 2014; Skrable & Fitzsimons, 2014; Tosterud, Hedelin, & Hall‐Lord, 2013; Tosterud, Petzall, Hedelin, & Hall‐Lord, 2014), researchers have paid comparatively little attention to identifying the elements in simulation that are associated with these positive outcomes. To our knowledge, only two studies have examined the associations between elements in scenario‐based simulation and students' self‐confidence and satisfaction as outcome measures (Smith & Barry, 2013; Smith & Roehrs, 2009). Smith and Roehrs (2009), who performed a simulation scenario involving an elderly patient with acute deterioration, found that two essential adult‐learning principles—having a clear statement of objectives and having opportunities for problem‐solving—were associated with high levels of student satisfaction and self‐confidence after simulation. In Smith and Barry's (2013) simulation of a homecare patient situation, learning principles, such as support and opportunities for problem‐solving, were found to be correlated with self‐confidence and satisfaction. Based on the results from these studies, several key elements are necessary to achieve successful simulation sessions: (a) having well‐defined and clear objectives for the simulation, (b) experiencing support during the simulation and (c) being provided with opportunities for problem‐solving that are adjusted to the students' level of knowledge.

Although Blum, Borglund, and Parcells (2010) found self‐confidence and competence to be poorly correlated, Lapkin et al. (2010) suggested that low levels of self‐confidence can have a detrimental effect on learning outcomes. Students may become better equipped for learning by gaining increased experience and self‐confidence (Najjar, Lyman, & Miehl, 2015; Yuan, Williams, Fang, & Ye, 2012). Levett‐Jones et al. (2011) have also suggested that student satisfaction helps to build self‐confidence, which in turn may help students to develop skills and acquire knowledge. Hence, to develop strategies to optimize students' learning outcomes, further studies are needed to identify which elements in simulation are related to student self‐confidence and satisfaction.

The aim of this study was to identify elements in scenario‐based simulation that are associated with nursing students' satisfaction with the simulation activity and their self‐confidence in managing the simulated patient situation. The study will provide insight to nursing educators to improve the use of simulation as a learning strategy. The simulation was implemented as a mandatory supplement to first‐year students' 6‐week clinical practice in nursing homes and aimed to merge theoretical knowledge, practical experiences and skills in a simulated situation where a ‘patient' experienced deterioration from a chronic disease.

## 3 METHODS

### 3.1 Study design, sample and setting

This cross‐sectional, observational study involved first‐year nursing students in the bachelor's degree programme in Norway, and rating scales were used for data collection. The reporting of the simulation session follows *Key Elements to Report for Simulation‐Based Research* (Cheng et al., 2016). The students (*N* = 202) were invited to participate in the study, and 187 volunteered after attending a 3‐hr simulation session held in the university college's skills laboratory. The students indicated their consent by anonymously filling out the questionnaire after the entire simulation session was completed.

At the time of the study, the students had completed their first clinical practice in nursing homes and had no former simulation experience. The level of fidelity in the scenario was considered as high due to the immersing of the students as autonomous clinicians making decisions and demonstrating their knowledge (Hamstra et al., 2014) and the use of clinical equipment and patient simulators (NursingAnne^®^; Laerdal™). The simulation and data collection were conducted in the spring of 2016, while data analysis was completed in 2018.

### 3.2 Data collection

The respondents were asked to assess the degree to which they agreed with various statements by using a five‐point Likert scale, where higher numbers indicated greater agreement. The questionnaires contained three instruments that a research team from the Norwegian University of Science and Technology in Gjøvik recently validated and translated into Norwegian: the Student Satisfaction and Self‐Confidence in Learning (SSSCL) scale, the Simulation Design Scale (SDS) and the Educational Practices Questionnaire (EPQ) scale (NLN, 2018). After Tosterud et al. (2013), Tosterud et al. (2014)) conducted a forward and back translation, Cronbach's alpha showed values above .8 for all translated instruments. The three instruments consist of 11 subscales that reflect the elements in the NLN Jeffries simulation theory (NLN, 2018).

The SSSCL scale is a 13‐item instrument that measures both students' self‐confidence in managing the simulated patient situation (eight items) and their satisfaction with the simulation activity (five items). Responses were provided on a five‐point Likert scale.

The SDS consists of 20 items, which include a five‐point Likert scale and ‘not applicable'. The SDS measures elements that are related to the simulation's design and to various adult‐learning principles, including:

- *Clear objectives*: the presence and importance of having clear and well‐defined objectives for the simulation session (five items).
- *Support*: the presence and importance of support and assistance from the facilitator during the simulation (four items).
- *Problem‐solving*: the presence and importance of opportunities to independently solve problems that are adjusted to the students' level of knowledge (five items).
- *Feedback*: the presence and importance of constructive feedback that increases knowledge (four items).
- *Fidelity (realism)*: the presence and importance of a real‐life situation with real‐life factors in the simulation scenario (two items).

The EPQ scale consists of 16 items, which also include a five‐point Likert scale and ‘not applicable'. The instrument measures elements related to the simulation's educational practices, including:

- *Active learning*: the presence and importance of active participation and opportunities to discuss ideas and concepts (ten items).
- *Collaboration*: the presence and importance of opportunities to work together with others during the session (two items).
- *Diverse ways of learning*: the presence and importance of opportunities to learn in various ways (two items).
- *High expectations*: the presence and importance of communicated objectives, goals and expectations (two items).

The original English versions of the three instruments are available for public use from the National League for Nursing (NLN, 2018).

### 3.3 The simulation session

**Figure 1:**
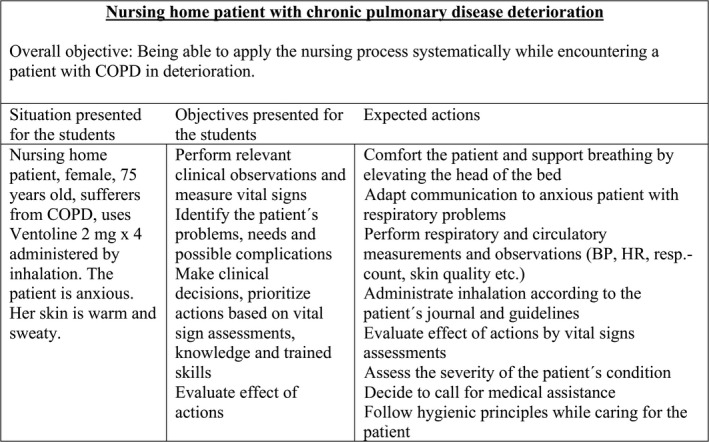
The simulation scenario and objectives

The NLN Jeffries simulation theory (Jeffries et al., 2015; Jeffries & Rogers, 2012) was used as a framework for designing and implementing the simulation session. The complexity of the scenario was adjusted to the students' curriculum, earlier classroom lectures and skills training and closely linked to an actual situation in a nursing home. A patient situation that is considered challenging to the students was chosen: a nursing‐home patient who experienced deterioration of a chronic obstructive pulmonary disease (COPD). The scenario required knowledge and skills in anatomy, physiology, pathophysiology, medication administration and nursing actions, as well as the ability to merge theoretical and practical knowledge to assess and act in accordance with the simulated patient's needs. The overall aim for the scenario was to apply the nursing process systematically while encountering a patient with COPD in deterioration (Figure 1).

The students were informed about objectives for the simulation session without disclosing the whole event or expected actions. They were told beforehand in a classroom lesson that they were expected to care for a nursing‐home patient with COPD by making clinical observations, decisions, actions and evaluations based on their knowledge and skills, and they were encouraged to prepare themselves by reading relevant literature. The students were also informed about the patient's age, gender and medical treatment as well as basic concepts related to simulation, such as confidentiality, conduct and expectations.

The 10 university college teachers who participated as facilitators were trained facilitators with experience in simulation‐based education and debriefing. Among the facilitators, six held formal facilitator education. The facilitators were given an instruction guide and rehearsed before the simulation to decrease the risk of variation in performance of the simulation sessions. The simulation sessions included groups of eight students and one facilitator. The facilitator was responsible for initial briefing, controlling the patient simulator with a control unit (Sim Pad^®^; Laerdal™) and facilitating the debriefing. Although making the simulators talk during the simulation was impossible because of a lack of proper simulation rooms, the acute patient situation that was chosen made it somewhat realistic that the patient had to concentrate on breathing rather than talking.

The simulation session consisted of a three‐step process: (1) an initial briefing (10–15 min); (2) simulation of the patient situation (15–20 min); and (3) debriefing (45–60 min). The initial briefing provided the students with an overview of the simulation steps, a repetition of the objectives and the ability to familiarize themselves with the surroundings, the patient simulator and the technical equipment (Jeffries & Rogers, 2012). In step 2, four students at a time participated as nurses in active, hands‐on simulation, while the remaining four were observers. The facilitators were instructed not to intervene, as they normally would in a nursing‐home environment, if students omitted specific types of care or made flawed clinical decisions. See Figure 1 for detailed description of the scenario and actions of nursing expected by students during the simulation. In step 3, the simulation was deconstructed and analysed in a structured debriefing that lasted for approximately 1 hr. After step 3, the whole session was run again with switched roles to allow all students to experience the role as nurses. The scenario remained unchanged. The active simulations were video recorded to enable the participants to observe and reflect on their actions during the debriefing. Recordings were deleted after the simulation sessions were completed.

The descriptive, analytic and application phase, described by Steinwachs (1992), was used as an approach to facilitate the debriefing. During the descriptive phase, the students were asked to describe what had happened in the situation, how they felt and what their principal challenges were. In the analytic phase, the students were encouraged to explore what they had done well and not so well, what decisions and actions they had made and why they had made these decisions. They were also challenged to analyse the situation theoretically and to explore parallels with real‐world situations. During the application phase, the students were asked to reflect on how they could improve their nursing care and decision‐making activities in future patient encounters as a result of their experiences and new understandings. The students were asked to fill out the questionnaires after the last debriefing session.

### 3.4 Data analysis

Participants' mean age and gender distribution were estimated based on the university college's public student register of enrolled first‐year students. Means and standard deviations (*SD*) were used to describe the dependent and independent variables. Multiple linear regression analyses were performed to examine the associations between elements in simulation design characteristics and educational practices and students' self‐confidence and satisfaction. The study had nine predictors, and the sample size of 187 was assumed to be large enough for regression analysis (Field, 2005). The regression analyses were performed by forced entry, meaning that all predictors were entered simultaneously. The method was based on theoretical reasoning, as the chosen predictors were elements drawn from a well‐known theoretical model (Field, 2005). Multicollinearity was taken into account when planning the multiple regression analyses and was tested through bivariate correlation analyses. The internal consistency of the scales was described by Cronbach's alpha values. Analysis was conducted using IBM SPSS Statistics, version 22.

### 3.5 Research ethics

One of the researchers also served as a teacher to the participating students. We ensured that the students were in an independent relationship with the researcher and that the researcher had no responsibilities to evaluate or grade the participants. The anonymous and voluntary nature of the students' participation was emphasized, and the students were informed about the study both orally and by email. The questionnaire required no background information or other sensitive material from the individual participants. The Norwegian Centre for Research Data (NSD, from Norsk senter for forskningsdata) was contacted for advice on the need for written informed consent; the NSD concluded that filling out the questionnaire implied informed consent.

## 4 RESULTS

The overall response rate was 92.6% (*N* = 187). According to the public student register of the university college, the mean age of the individuals enrolled as first‐year students was 24.21 years (*SD* 2.96) and 10% were male. The mean SSSCL scale score was 4.32 (Table 1), and internal consistency for the scale was .783 (Cronbach's alpha).

**Table 1:** Mean SSSCL scale scores (*N* = 187)

	Mean (*N*)	*SD*
**Student satisfaction and self‐confidence in learning (overall)**	**4.32 (187)**	**0.34**
Satisfaction with their current learning	4.57 (187)	0.44
Self‐confidence in their learning	4.16 (187)	0.39

The bold text and values are the overall SSSCL score.

The SDS and EPQ scores were 4.54 and 4.50, respectively. The students' mean score for the importance of both items was higher than 4 (Table 2). For all independent variables, the Cronbach's alpha values were above .7 (*simulation design characteristics*: .859 for the presence of elements and .912 for their importance; *educational practices*: .795 for the presence of elements and .859 for their importance).

**Table 2:** Mean score of students' responses to SDS and EPQ (*N* = 187)

	Presence of items		Importance of items	
	Mean (*N*)	*SD*	Mean (*N*)	*SD*
**Simulation design characteristics (overall)**	**4.54 (184)**	**0.38**	**4.58 (182)**	**0.42**
Clear objectives	4.44 (184)	0.53	4.51 (182)	0.52
Support	4.54 (184)	0.55	4.55 (180)	0.57
Problem‐solving	4.39 (184)	0.55	4.50 (180)	0.53
Feedback/guided reflection	4.73 (183)	0.41	4.71 (180)	0.47
Fidelity (realism)	4.82 (183)	0.39	4.83 (178)	0.40
**Educational practices questionnaire (overall)**	**4.50 (185)**	**0.34**	**4.43 (180)**	**0.42**
Active learning	4.39 (184)	0.41	4.34 (177)	0.49
Collaboration	4.90 (184)	0.26	4.68 (179)	0.55
Diverse ways of learning	4.55 (184)	0.54	4.52 (178)	0.55
High expectations	4.58 (184)	0.60	4.54 (178)	0.60

The bold text and values are the overall SDS an EPQ scores.

The dependent variables (satisfaction and self‐confidence) and independent variables (active learning, collaboration, diverse ways of learning, high expectations, clear objectives, support, problem‐solving, feedback and fidelity) were modestly skewed towards the right but were considered normally distributed. Multicollinearity was not considered a problem, since the correlation coefficients between the independent variables were below .6 in bivariate correlation analyses. Since the chosen predictors were elements drawn from a well‐known theoretical model, we performed the multivariate regression analyses by forced entry of all independent variables. In the multivariate regression analysis with *satisfaction* as the dependent variable, the independent variable active learning explained 35.3% of the variance (*R* ^2^ = .35, Adjusted *R* ^2^ = .35, *F* = 11.96, *p *< .001). Active learning was significantly associated with satisfaction (Table 3).

**Table 3:** Multivariate regression: associations between independent variables and satisfaction (*N* = 187)

	Adjusted				Unadjusted
	Regression coefficient (*p*)	*SE*	Confidence interval		Regression coefficient (*p*)
**Active learning**	**.28 (<.001)**	**0.59**	0.13	0.49	.30 (<.001)
Collaboration	−.05 (.410)	0.09	−0.30	0.12	−.09 (.410)
Diverse ways of learning	.11 (.133)	0.11	−0.03	0.21	−.09 (.133)
High expectations	.03 (.741)	0.06	−0.09	0.13	.02 (.741)
Clear objectives	.08 (.319)	0.06	−0.07	0.21	.07 (.319)
Support	.12 (.130)	0.07	−0.03	0.23	.10 (.130)
Problem‐solving	.08 (.360)	0.07	−0.08	0.21	.07 (.360)
Feedback	.07 (.348)	0.09	−0.09	0.25	.08 (.348)
Fidelity	.11 (.103)	0.08	−0.03	0.28	.13 (.103)

The statistically significant values are written bold (*p *< .05).

The analysis was repeated with active learning as the only independent variable. The results showed that 27.8% (*R* ^2^ = .28) of the variance in satisfaction was explained by this element. In multivariate regression analysis with *self‐confidence* as the dependent variable, 30.8% of the variance was explained by three of the independent variables (*R* ^2^ = .31, Adjusted *R* ^2^ = .31, *F* = 9.96 *p *< .001). Experiencing clear objectives, support and opportunities for active learning were significantly associated with self‐confidence. The experience of having less support from facilitators resulted in higher self‐confidence (Table 4).

**Table 4:** Multivariate regression: associations between independent variables and self‐confidence (*N* = 187)

	Adjusted				Unadjusted
	Regression coefficient (*p*)	*SE*	Confidence interval		Regression coefficient (*p*)
**Active learning**	**.30 (<.001)**	**0.08**	0.13	0.43	.28 (<.001)
Collaboration	.04 (.520)	0.09	0.12	0.24	.06 (.520)
Diverse ways of learning	.13 (.095)	0.05	−0.02	0.19	.09 (.095)
High expectations	.06 (.481)	0.05	−0.06	0.13	.03 (.481)
**Clear objectives**	**.29 (.001)**	**0.06**	0.09	0.33	.21 (.001)
**Support**	**−.18 (.038)**	**0.06**	−0.23	−0.01	−.12 (.038)
Problem‐solving	.02 (.799)	0.06	−0.11	0.14	.02 (.799)
Feedback	.05 (.501)	0.08	−0.01	0.20	.05 (.501)
Fidelity	.09 (.222)	0.07	−0.05	0.22	.08 (.222)

The statistically significant values are written bold (*p *< .05).

The multivariate regression analysis was repeated, with clear objectives, support and active learning as the independent variables; the analysis showed that 28.6% (*R* ^2^ = .29) of the variance in self‐confidence was explained by these elements. Active learning and clear objectives were positively associated with self‐confidence, explaining 28.1% (*R* ^2^ = .29) of the variance, while the subscale support was not significantly associated with self‐confidence in this part of the analysis.

## 5 DISCUSSION

Most students felt self‐confident and were satisfied with the simulation activity. We found that active learning is important to attain self‐confidence and student satisfaction and learning objectives for the simulation were positively associated with self‐confidence. The students' needs for support were negatively associated with self‐confidence.

The positive evaluations regarding student satisfaction and self‐confidence found in the present study are in line with the results of previous studies (Cant & Cooper, 2010, 2017; Haddeland et al., 2018; Smith & Barry, 2013; Wotton, Davis, Button, & Kelton, 2010). Student satisfaction is an important outcome in education, because it may enhance students' engagement and thereby facilitate learning and, ultimately, the nursing students' competency and the quality of care provided by them (Levett‐Jones et al., 2011). Students' self‐confidence and satisfaction are probably insufficient to assess or evaluate learning or the overall impact of simulation (Jeffries & Rogers, 2012), although having knowledge about elements that are associated with students' self‐confidence and satisfaction may be essential in the development of effective and immersive scenario‐based simulation in nursing education (Prion, 2008).

Our results indicate that the presence of active learning contributed to both student satisfaction with the simulation activity and self‐confidence in managing the simulated patient situation. The relationship between active learning and satisfaction and self‐confidence may be explained from the social constructivism perspective, according to which learning is constructed in environments where students can actively interact with others (Vygotsky, 1978). In simulation, active learning and collaboration are inherent features and students have the opportunity to actively engage by using their whole body, their cognitive assets and their psychological and interactional skills to help the ‘patient'. Collaboration was not significantly associated with satisfaction and self‐confidence in this study, but collaboration promotes learning by opportunities to work together to solve problems, mimicking what is actually done in real life (Jeffries, 2005).

The fidelity variable refers to how authentic or life‐like the simulation experience is, but also on how the students are engaged in the situation (Hamstra et al., 2014). In the present study, we immersed the students by having them all actively perform hands‐on simulation, collaborating as both nurses and observers, as recommended in other studies (Leigh, 2008; Thidemann & Söderhamn, 2013; Tosterud et al., 2013). To further immerse the students and promote elements of the NLN Jeffries simulation theory, such as providing diverse ways of learning and feedback, the facilitators were instructed to ensure that all the students also contributed actively during the debriefing step (Jeffries & Rogers, 2012). Although the feedback variable was not associated with students' satisfaction and self‐confidence in our study, debriefing will most likely provide constructive feedback from fellow students and facilitators as described in previous studies (Levett‐Jones & Lapkin, 2014). Active student engagement during the simulation and debriefing sessions also accommodate diverse ways of learning and allow students with varying backgrounds to benefit from the experience (Jeffries, 2005). Our emphasis on active learning when planning the simulation activity is supported by Adamson, Jeffries, and Rogers (2012) who state that educators who prioritize active engagement in every step of the simulation activity may at the same time enhance the presence of other elements in design and educational practices. This statement may account for why our results showed no statistically significant associations with several of the elements. Active learning may be an overreaching variable that is experienced as most essential for the students. Our results do not indicate that educators should pay less attention to other elements but rather that active learning should be properly addressed as part of all elements in the development and implementation of simulation activities.

We provided all first‐year students equal opportunities for active learning during the simulation. This offer was highly demanding in terms of resources and time. For such reasons, it is challenging for educators to implement fully immersed simulations that emphasize student engagement without affecting other content of the curriculum. Faculties may try to solve resource‐related issues by providing the active hands‐on simulation for only a portion of the students, while assigning most of the students to be observers. Students who are only assigned the role of observer may disengage from the learning process, although at least one study has shown that being an active observer provides learning opportunities in each simulation step (Hober & Bonnel, 2014), Thidemann and Söderhamn (2013) found that students who were assigned the nurse's role in simulations were more self‐confident and satisfied than students who were assigned other roles (such as physicians) or were merely observers.

On the other hand, high expectations of active student engagement and performance in simulation may promote anxiety among some students (Al‐Ghareeb, Cooper, & McKenna, 2017; Jeffries & Rogers, 2012). Such anxiety has been identified as a universal experience of students who participate in simulations, but can be so overwhelming that it reduces self‐confidence and inhibits cognitive processing and the ability to apply knowledge (Al‐Ghareeb et al., 2017; Najjar et al., 2015; Nielsen & Harder, 2013). At the same time, a certain level of anxiety and reduced self‐confidence may also lead to excellent performance and can enhance students' motivation to engage in simulations (Al‐Ghareeb et al., 2017). Learning implies moving out of one's comfort zone, and in simulation activities, students are expected to perform while others watch their steps. The anxiety, tension and occasional frustration that students experience may be a necessary prerequisite for learning. Educators should still bear in mind that excessive levels of anxiety may negatively influence knowledge acquisition and diminish performance and they should place emphasis on creating an atmosphere where students feel safe (Al‐Ghareeb et al., 2017; Dieckmann, Friis, Lippert, & Østergaard, 2012; Leigh, 2008; Nielsen & Harder, 2013). As our findings showed that active learning was associated with self‐confidence and satisfaction, it is indicated that the anxiety level was not too high. Even though the active learning element was emphasized in the design of this simulation session, we also had the safety of the students in mind. However, the quantitative design of the present study makes it difficult to reveal whether single students have experienced the simulation session as negative in terms of anxiety level.

A major principle inherent in adult learning is to promote the students' understanding of their learning needs (Knowles, Holton, & Swanson, 1998). According to Lioce et al. (2015), students should know the objectives for the simulation activity without knowing all the challenges they will meet in the scenarios. In the NLN Jeffries simulation theory, it is described that the students' opportunities to solve problems should be adjusted to the student's level of knowledge (Jeffries & Rogers, 2012). However, it is highlighted by Lindsey and Berger (2009) that this adjustment should not be at the expense of the students' experience of challenge (Lindsey & Berger, 2009). Opportunities for problem‐solving and clear objectives for the simulation session may allow the students to perform the simulation successfully, but problem‐solving was not found to be associated with satisfaction and self‐confidence in the present study (Lindsey & Berger, 2009; Wilson & Klein, 2012). However, the association between clear objectives and self‐confidence was identified and is supported by several authors who emphasize the importance of developing clear and well‐defined objectives for simulation sessions to enhance learners self‐confidence (Jeffries & Rizzolo, 2006; Smith & Roehrs, 2009; Wilson & Klein, 2012). Self‐confidence may affect students' ability to engage in critical reflection as well as their efforts and persistence when confronted with challenges in practice (Bandura, 1997). Dieckmann et al. (2012) have underlined the necessity of a shared understanding of the objectives of a simulation session, but specific performance objectives or scenario events should not be presented for the learners prior to the simulation (Lioce et al., 2015). If the scenario is ‘given away' before it starts, the students' opportunities to learn and to recognize when they need to apply prior learning is decreased. Lioce et al. (2015) highlight that only those objectives that provide general information and context for the learner should be disclosed prior to the simulation. Thus, developing clear and well‐defined objectives for the simulation (Jeffries et al., 2015; Jeffries & Rogers, 2012) does not necessarily mean that educators should make the specific performance objectives available for the students beforehand. Rather, the educators should guide the students towards reaching the specific objectives during the simulation session (Lioce et al., 2015). The results of the present study indicate that our efforts to inform and prepare the students about their ‘need to know' were successful. However, it is difficult to know where the boundary between too much and too little information goes and this issue should be discussed by a team of educators prior to performance of simulation sessions.

We found that low scores on the support variable were associated with higher levels of self‐confidence. One explanation for this situation may be that the students experienced that the objectives were expressed in such a way that a balance was created between independent and active participation and challenges. This explanation also indicates that educational practices and design elements are interwoven and that all elements should be addressed in the design of simulation sessions. Another explanation may be that the students' expectations of self‐direction and responsibility for their own learning prior to the simulation session were high and that they may have experienced intervention of facilitators as disturbing (Jeffries & Rogers, 2012). Even though the facilitators were instructed not to intervene if the students omitted specific types of care or made poor clinical decisions during the simulation, it is difficult to rule out that facilitators interpret the instructions differently. According to Jeffries and Rogers (2012), assistance should not interfere with the students' problem‐solving efforts because students may act more passively during learning situations (Knowles et al., 1998). The excessive offering of support may thus inhibit learning and affect the students' evaluation of that support.

## 6 LIMITATIONS

A cross‐sectional design was suitable for this study, as it did not aim to prove causality but to describe the associations between elements in the simulation session and the students' self‐confidence and satisfaction. Because the questionnaire contained both outcome variables and independent variables, the presence of common method bias cannot be ruled out, although we do not believe that the use of more than one method would have altered the results.

The lack of control group in our study makes it difficult to decide whether the students' high levels of satisfaction and self‐confidence were a result of the scenario‐based simulation, the first‐year students' novel experience with simulation, or their general self‐confidence and satisfaction with their education. Conducting research in one's own organization can potentially raise issues of an imbalance of power between the inquirers and the participants (Creswell, 2014). The presence of faculty teachers during the simulation may have shaped the way the students answered the questionnaires. Due to the lack of diversity in terms of context and participants, one should be careful about generalizing the results of the present study. We did not obtain individual characteristics of the participants and were therefore unable to adjust for individual characteristics. We were only able to present the age and gender distribution of all students enrolled as first‐year students in the student registry.

## 7 CONCLUSIONS

The findings of the present study indicate that opportunities for active learning and conveying learning objectives for the simulation session should be emphasized in the development and implementation of simulation activities. Active learning may increase both student satisfaction with the learning activity and self‐confidence in managing the simulated patient situation and educators should be particularly concerned with providing opportunities for active participation in the learning process. While educators should pay attention to all elements in the NLN Jeffries simulation theory to develop a successful simulation experience, we suggest that emphasizing active learning and objectives may have an essential impact on the other elements of educational practices and simulation design.

## CONFLICT OF INTERESTS

The authors declare that they have no competing interests.

## AUTHORS' CONTRIBUTIONS

CO was responsible for the conception, design, analysis and interpretation of data and worked out the drafts and completed the submitted version of the manuscript. KH and CRT participated in the analyses and interpretation of the data and contributed to the manuscripts intellectual content and critical review. All authors have given their final approval for the submitted version.

## ETHICS APPROVAL AND CONSENT TO PARTICIPATE

The NSD was contacted for advice on the need for written informed consent; the centre concluded that filling out the questionnaire was considered informed consent. The questionnaires did not have any identity numbers or codes.
